# Activity of newest generation β-lactam/β-lactamase inhibitor combination therapies against multidrug resistant *Pseudomonas aeruginosa*

**DOI:** 10.1038/s41598-022-21101-x

**Published:** 2022-10-07

**Authors:** Robbie R. Haines, Papanin Putsathit, Katherine A. Hammer, Anna S. Tai

**Affiliations:** 1 School of Biomedical Sciences, The University of Western Australia, 30 Stirling Hwy, Crawley, Perth, WA 6009 Australia; 2 School of Medical and Health Sciences, Edith Cowan University, Joondalup, WA Australia; 3 Department of Respiratory Medicine, Sir Charles Gairdner Hospital, Nedlands, WA Australia; 4 Institute of Respiratory Health, Nedlands, WA Australia; 5 Medical School, The University of Western Australia, Perth, WA Australia

**Keywords:** Antibiotics, Antimicrobial resistance, Bacteriology, Infectious-disease diagnostics

## Abstract

Multidrug resistant (MDR) *P. aeruginosa* accounts for 35% of all *P. aeruginosa* isolated from respiratory samples of patients with cystic fibrosis (CF). The usefulness of β-lactam antibiotics for treating CF, such as carbapenems and later generation cephalosporins, is limited by the development of antibacterial resistance. A proven treatment approach is the combination of a β-lactam antibiotic with a β-lactamase inhibitor. New β-lactam/β-lactamase inhibitor combinations are available, but data are lacking regarding the susceptibility of MDR CF-associated *P. aeruginosa* (CFPA) to these new combination therapies. In this study we determined MIC values for three new combinations; imipenem-relebactam (I-R), ceftazidime-avibactam (CZA), and ceftolozane-tazobactam (C/T) against MDR CFPA (n = 20). The MIC_90_ of I-R, CZA, and C/T was 64/4, 32/4, and 16/8 (all µg/mL), respectively. The susceptibility of isolates to imipenem was not significantly improved with the addition of relebactam (p = 0.68). However, susceptibility to ceftazidime was significantly improved with the addition of avibactam (p < 0.01), and the susceptibility to C/T was improved compared to piperacillin/tazobactam (p < 0.05) These data provide in vitro evidence that I-R may not be any more effective than imipenem monotherapy against MDR CFPA. The pattern of susceptibility observed for CZA and C/T in the current study was similar to data previously reported for non-CF-associated MDR *P. aeruginosa.*

## Introduction

Cystic fibrosis (CF) is a genetic disease affecting the osmotic control of airway mucus. The resulting mucus is thick and tenacious, which impairs the mucociliary elevator, obstructs the flow of air, and impedes immune effectors from reaching the airways efficiently^1^. As a result of these disease features, bacterial infection is common in the CF patient population^1^. *Pseudomonas aeruginosa* is responsible for the majority of bacterial lung infections in the adult CF population; in 2019 77.6% of Australian CF patients older than 25 years had *P. aeruginosa* present in their respiratory tract^2^. Colonisation and infection with CF-associated *P. aeruginosa* (CFPA) are significant events in the clinical course of the CF, and are associated with increased morbidity and mortality^3^.

*P. aeruginosa* has intrinsic resistance to many antimicrobials and also has a propensity to both develop and acquire antimicrobial resistance (AMR) genes through mutation and horizontal gene transfer, respectively^4^. One method by which resistance may develop to β-lactam antibiotics in particular is by horizontal gene transfer of genes encoding β-lactamases^5^. In the twentieth century, a major clinical breakthrough was the discovery of compounds that bind to β-lactamases and inhibit their activity. These compounds (namely tazobactam, sulbactam, and clavulanic acid) decrease the effective rate of hydrolysis of β-lactam antibiotics by some β-lactamases, thereby renewing antimicrobial action against bacteria that relied on those enzymes for resistance^6,7^.

Unfortunately, the range of β-lactamases that these original three β-lactamase inhibitors can act upon is limited, causing an evolutionary pressure for these enzymes to mutate and become inhibitor resistant^7^. As with all AMR, there is an artificial selection pressure created leading to the proliferation of inhibitor-resistant β-lactamases^7^. Generally speaking, tazobactam/sulbactam/clavulanic acid are active against class A and class D serine β-lactamases, but not class B metallo-β-lactamases or class C serine β-lactamases^7^. Recent innovations have led to the development and licencing of new β-lactamase inhibitors that have activity against all serine β-lactamase (classes A, C, and D)^8^. The three new non-β-lactam β-lactamase inhibitors currently available are vaborbactam (with meropenem), avibactam (with ceftazidime), and relebactam (with imipenem [IPM] and cilastatin). Also, another recent innovation has led to the synthesis of a new cephalosporin with anti-pseudomonal activity, ceftolozane, which is available in combination with tazobactam.

Prior epidemiological and in vitro studies suggest that ceftazidime/avibactam (CZA) and ceftolozane/tazobactam (C/T) are effective therapies against β-lactam resistant *P. aeruginosa* (61.8–70.2%, and 72.5–78.7% of isolates susceptible, respectively)^9,10^. Meropenem/vaborbactam is active against narrower range of MDR *P. aeruginosa* isolates (59.0%)^11^. Imipenem/relebactam (I-R) is a more recent therapy and therefore the data are limited, however, some data suggest that the addition of relebactam may not be sufficient to lower the MIC of IPM below a clinically useful level in *P. aeruginosa* that produce β-lactamases^12^.

The high prevalence of multidrug resistant (MDR) CFPA is of particular concern and accounts for 35% of all CFPA in the Australian adult CF patient population^13^. New β-lactam/β-lactamase inhibitor combinations are available, but the in vitro efficacy of these new combinations is not well established against CFPA isolates.

CFPA isolates have a different distribution of genotypes compared to *P. aeruginosa* isolates not originating from CF^14^. Due to these genetic differences and the unique physiological conditions present in the CF airway, atypical phenotypes such as mucoid variants and small colony variants have emerged^15–17^. These macroscopic phenotypic changes are evidence of other more subtle changes in the phenome, some of which may include acquisition/expression of β-lactamases. Due to the phenotypic divergence of CFPA from non-CFPA, the efficacy of antimicrobials against this group must be verified. In this study we determined the MIC of I-R, CZA, and C/T against MDR CFPA isolates.

## Results

MICs of I-R, CZA, and C/T for CFPA isolates (n = 20) are shown in Table 1. Of particular note are the MIC_50_ values for CZA and C/T which fall below the Clinical and Laboratory Standards Institute (CLSI) breakpoints that indicate susceptibility to those agents. Twenty percent (n = 3/15) of IPM resistant isolates were I-R susceptible, 68.8% (n = 11/16) of ceftazidime (CAZ) resistant isolates were CZA susceptible, and 68.8% (n = 11/16) of piperacillin/tazobactam (TZP) resistant isolates were C/T susceptible. Complete results for each isolate are shown in Table 2, and full sensitivities at the screening stage are showing in Supplementary Table S1. The addition of relebactam to IPM did not significantly change resistance to IPM alone (p = 0.6831), however the addition of avibactam to CAZ significantly altered resistance (p = 0.0094). The change from TZP to C/T had a significant effect on resistance phenotype (p = 0.0265). Some strains showed some level of phenotypic variance in that they would be sensitive to the single agent, but become phenotypically resistant when the inhibitor was added (e.g.: CFPA 04).

**Table 1:** MIC values for CFPA isolates (n = 20). All values are in µg/mL.

Test agent	MIC_50_	MIC_90_	Range	CLSI breakpoints		
				**S**	**I**	**R**
I-R	32/4	64/4	< 0.5/4–128/4	≤ 2/4	4/4	≥ 8/4
CZA	8/4	32/4	< 1/4–> 512/4	≤ 8/4	–	≥ 16/4
C/T	2/1	16/8	< 0.5/0.25–64/32	≤ 4/4	8/4	≥ 16/4

**Table 2:** Genotypes and AMR phenotypes of isolates used in this study.

Isolate	Possible MLST STs (strain)^a^	IPM^b^	I-R^c^	CAZ^b^	CZA^c^	TZP^b^	C/T^c^
CFPA 01	649 **(AUST-01)**	R	R	R	S	R	S
CFPA 02	649 **(AUST-01)**	R	R	R	S	R	S
CFPA 03	775 **(AUST-02)**	R	R	S	R	S	R
CFPA 04	775 **(AUST-02)**	S	R	R	S	R	S
CFPA 05	775 **(AUST-02)**	S	S	R	S	R	S
CFPA 06	242 **(AUST-03)**, 996	S	R	R	R	R	R
CFPA 07	242 **(AUST-03)**, 996	R	R	R	R	R	S
CFPA 08	787, 788 **(AUST-04)**	R	S	R	S	S	S
CFPA 09	4, 801 **(AUST-06)**, 1292	R	R	R	R	R	R
CFPA 10	4, 801 **(AUST-06),** 1292	R	R	R	R	R	R
CFPA 11	262 **(AUST-07)**, 774, 1165	R	R	S	S	S	R
CFPA 12	262 **(AUST-07)**, 774, 1165	S	R	S	S	S	S
CFPA 13	882 **(AUST-11)**, 1151, 1233	R	S	S	S	R	S
CFPA 14	882 **(AUST-11)**, 1151, 1233	S	S	R	S	R	S
CFPA 15	905 **(AUST-16)**, 1039	R	R	R	S	R	S
CFPA 16	905 **(AUST-16)**, 1039	R	R	R	R	R	R
CFPA 17	12 **(AUST-33)**	R	R	R	S	R	S
CFPA 18	254 **(AUST-38)**, 1041 **(AUST-38)**	R	R	R	S	R	S
CFPA 19	217 **(Manchester)**, 1134	R	S	R	S	R	S
CFPA 20	217 **(Manchester)**, 1134	R	R	R	S	R	R
ATCC® 27,853	115	S	S	S	S	S	S
PAO1	549	S	S	S	S	S	S
	Total susceptible^d^ (%) (n = 20)	25	25	20	70	20	65

*IPM* imipenem, *I-R* imipenem/relebactam, *CAZ* ceftazidime, *CZA* ceftazidime/avibactam, *TZP* piperacillin/tazobactam, *C/T* ceftolozane/tazobactam.

^a^Genotypes (MLST STs) associated with the iPLEX20SNP profile. Named strains associated with genotypes indicated in bold.

^b^Susceptibility determined by disk diffusion assay.

^c^Susceptibility determined by broth microdilution assay.

^d^Excludes reference strains *P. aeruginosa* ATCC® 27,853 and *P. aeruginosa* PAO1.

## Discussion

In this study we have demonstrated the in vitro antimicrobial efficacy of three new antipseudomonal treatments, I-R, CZA and C/T, against CFPA isolates.

Only a modest proportion of IPM resistant isolates were I-R susceptible. This suggests that relatively few isolates had class A or class C β-lactamase-mediated IPM resistance. This is an expected result, as the majority of IPM resistance is mediated by changes in membrane permeability associated with inactivation of the efflux pump *oprD*, which is unaffected by β-lactamase inhibitors, including relebactam^18^. I-R may be useful in infections caused by the subset of CFPA isolates that have IPM resistance mediated by class A or class C β-lactamases only. Relebactam does not have class B metallo-β-lactamase or class D serine β-lactamase activity, and therefore I-R should not be considered when these enzymes are either suspected or confirmed to be a resistance mechanism in the target isolate^12^.

Our results are discordant with I-R susceptibility data for non-CFPA isolates, which indicate that approximately 80% of MDR isolates are susceptible to I-R^19^. β-lactamase-mediated resistance is more common in non-CFPA and non-β-lactamase mechanisms such as *oprD* inactivation are responsible for the some IPM resistance in MDR CFPA^20^.

The majority of CAZ resistant isolates were CZA susceptible, which is consistent with the literature for MDR non-CFPA isolates^9,10^. The relatively high proportion of CAZ resistant isolates that were also CZA susceptible suggests that the majority of CAZ resistance is mediated primarily by serine β-lactamases which have interactions with avibactam. Avibactam is known to interact strongly with class A, class C, and some class D β-lactamases^21^.

Piperacillin is used as a comparator to ceftolozane given that they both share tazobactam as a combination agent, and also have similar uses in the clinic in the context of *P. aeruginosa*. Predictably, the majority of TZP resistant isolates were C/T susceptible, demonstrating that the newest generation cephalosporin, ceftolozane, has a wider spectrum of activity compared to the ureidopenicillin piperacillin.

The proportions of MDR CFPA isolates in this study that were susceptible to CZA, and C/T are consistent with values reported in the literature for MDR non-CFPA^9,10,12^. This suggests that for CZA and C/T there is not yet a divergent resistance phenotype in CFPA, and that research to-date involving MDR *P. aeruginosa* and these agents can be broadly applied to MDR CFPA. Data from both the current and previous studies should be interpreted with some measure of caution as none of the conventionally used antibiotic susceptibility testing methods (disk diffusion assay, agar dilution assay, broth microdilution assay) account for the dynamic in vivo environment. One such consideration is the presence of biofilm. *P. aeruginosa* is a biofilm-forming organism, and the mature biofilm of *P. aeruginosa* confers tolerance to antimicrobials by virtue of limiting drug diffusion^22^. This is an important determinant of AMR and must always be considered in the treatment of *P. aeruginosa* infections^22,23^.

Having determined whether an organism is susceptible to an antimicrobial agent, the effective delivery of that antimicrobial must also be considered. Due to the altered whole body systems physiology present in CF patients, antimicrobial pharmacokinetics/pharmacodynamics are affected^24^. A pertinent example is the enhanced renal (and total) clearance of β-lactam antibiotics in the CF patient population^24^. Due to this niche pharmacokinetic difference, subinhibitory concentrations may develop quicker during intermittent dosing regimens in the CF patient population. Therapeutic drug monitoring and/or continuous dosing can be used to address this problem^24,25^.

Whilst this study provides important in vitro data supporting the clinical use of new combinations, it is important that the potential for MDR CFPA to develop resistance to these combinations also be evaluated. The CFPA isolates that were used in this study were naïve to I-R, CZA, and C/T, and there has been no opportunity allowed for our isolates to develop mutations conferring resistance on any meaningful timescale. However, it is clear that there was short-term variations in resistance phenotype. This is exemplified by resistance to a combination therapy, but susceptibility to the monotherapy. This may be an on-going limitation when performing in vitro susceptibility testing on hypermutative bacteria such as CFPA. Additional studies examining the propensity for organisms to develop and propagate resistance to these antimicrobial agents is needed at both the laboratory and population health scale, especially considering resistance mechanisms against each of the β-lactam/β-lactamase inhibitor combinations in this study have been identified and reported in the literature^26–28^.

Our results indicate a high proportion of CFPA isolates that are resistant to IPM are also resistant to I-R, however CAZ resistant isolates may be susceptible to CZA, and TZP resistant isolates may be susceptible to C/T. Where possible, antibiotic susceptibility testing should always be used to identify appropriate antimicrobial agents, and good prescribing practices should continue to reflect this important component of antimicrobial stewardship.

## Materials and methods

### Experimental isolates

Sputum samples were collected at the Western Australian Adult Cystic Fibrosis Centre between January 2017 and May 2018. All samples were cultured for *P. aeruginosa*. Collection of sputum samples was approved by the Sir Charles Gairdner Hospital Human Research Ethics Committee (SCGH HREC RGS 0000001815) and in accordance with institutional policies and procedures; informed consent was obtained from all patients. Any *P. aeruginosa* strains isolated subsequently underwent antimicrobial susceptibility testing via the disk diffusion assay against meropenem, IPM, TZP, cefepime, CAZ, aztreonam, colistin, tobramycin, gentamicin, amikacin, ciprofloxacin, and levofloxacin. The disk diffusion assay was performed and interpreted according the CLSI guidelines^29,30^. Based on these results, isolates were categorised as non-MDR or MDR using consensus criteria^31^. The genotype of each isolate was determined using iPLEX20SNP, a validated method using MassARRAY MALDI-TOF MS which is described elsewhere^32^. All isolates included in this study were obtained from samples provided by unique patients and were obtained on separate clinic visits. For these reasons, isolates with identical iPLEX20SNP profiles were considered unlikely to be clonal. *P. aeruginosa* ATCC® 27853 was included as a quality control organism and *P. aeruginosa* PAO1 was included as a comparator.

### Determination of MICs

A broth microdilution assay was used to determine the MICs of I-R, CZA, and C/T. The procedure followed is described in the CLSI standard, and the results were interpreted using the CLSI published breakpoints, shown in Table 1^30,33^. MICs were determined visually and verified by measuring the OD_600_ of each well of the microplate before and after incubation. A ≥ 90% reduction in OD_600_ compared to the positive growth control was considered to be inhibitory and was the method used to determine MIC values. The spectrophotometric method corresponded well with the results obtained visually.

### Antimicrobial agents

CAZ, IPM, relebactam, and avibactam (item numbers HY-B0593, HY-B1369, HY-16752, and H-14879A) were purchased from MedChemExpress. Ceftolozane was not available as a single agent, and therefore the clinical formulation of C/T (ARTG 229608) was used. Since this formulation contained ceftolozane in a fixed concentration of 2:1 with tazobactam (1 g/0.5 g), this represents a variance form the CLSI protocol which uses a tazobactam concentration of 4 µg/mL in all microplate test wells^30,34^.

### Statistical analyses

All MIC values were obtained in biological triplicates for each strain and the modal value was reported as the MIC value in this study. For the purposes of analysis, isolates that had MICs falling in the intermediate breakpoint range were reported as resistant. McNemar’s test was performed to test for statistical significance between the resistance of IPM and I-R, CAZ and CZA, TZP and C/T. P values less than 0.05 were considered significant. McNemar’s tests were performed using the free browser tool QuickCalcs provided by GraphPad Software LLC (https://www.graphpad.com/quickcalcs/).

## Supplementary Information

Supplementary Information.

## Data Availability

All data generated is contained within the tables published.
