# Supplementary material for: Activity of newest generation β-lactam/β-lactamase inhibitor combination therapies against multidrug resistant Pseudomonas aeruginosa
Source: Sci Rep. 2022 Oct 7;12:16814. doi: 10.1038/s41598-022-21101-x (PMC9547053; doi:10.1038/s41598-022-21101-x)
Supplement: Supplementary file 1 — Supplementary Information. [file 41598_2022_21101_MOESM1_ESM.docx]

**Supplementary Table 1** AMR phenotypes of isolates used in this study as determined by disk diffusion assay.

| **Isolate** | **MEM** | **IPM** | **ATM** | **CST** | **TOB** | **FEP** | **CAZ** | **AMK** | **TZP** | **CIP** | **GEN** | **LVX** |
| --- | --- | --- | --- | --- | --- | --- | --- | --- | --- | --- | --- | --- |
| CFPA 01 | R | R | R | S | R | R | R | R | R | R | R | R |
| CFPA 02 | S | R | R | S | R | R | R | R | R | R | R | R |
| CFPA 03 | S | R | S | R | R | R | S | R | S | R | R | R |
| CFPA 04 | S | S | R | S | R | R | R | R | R | R | R | R |
| CFPA 05 | S | S | R | S | R | R | R | R | R | R | R | R |
| CFPA 06 | S | S | R | S | R | S | R | R | R | R | R | R |
| CFPA 07 | R | R | R | S | R | R | R | R | R | R | R | R |
| CFPA 08 | S | R | R | S | R | R | R | R | S | R | R | R |
| CFPA 09 | R | R | R | S | R | R | R | R | R | R | R | R |
| CFPA 10 | R | R | R | S | R | R | R | R | R | R | R | R |
| CFPA 11 | S | R | S | S | R | S | S | R | S | R | R | R |
| CFPA 12 | S | S | S | S | R | S | S | R | S | R | R | R |
| CFPA 13 | S | R | R | S | R | R | S | R | R | R | R | R |
| CFPA 14 | S | S | R | S | R | R | R | R | R | R | R | R |
| CFPA 15 | R | R | R | S | R | R | R | R | R | R | R | R |
| CFPA 16 | R | R | R | S | R | R | R | R | R | R | R | R |
| CFPA 17 | R | R | R | S | R | R | R | R | R | S | R | S |
| CFPA 18 | S | R | R | S | R | R | R | R | R | R | R | R |
| CFPA 19 | R | R | R | S | R | R | R | R | R | S | R | R |
| CFPA 20 | R | R | R | S | R | R | R | R | R | R | R | R |
| Total susceptible (%) (n=20) | 55 | 25 | 15 | 95 | 0 | 15 | 20 | 0 | 20 | 10 | 0 | 5 |
